# Assessment of atmospheric emissivity models for clear-sky conditions with reanalysis data

**DOI:** 10.1038/s41598-023-40499-6

**Published:** 2023-09-02

**Authors:** Luis Morales-Salinas, Samuel Ortega-Farias, Camilo Riveros-Burgos, José L. Chávez, Sufen Wang, Fei Tian, Marcos Carrasco-Benavides, José Neira-Román, Rafael López-Olivari, Guillermo Fuentes-Jaque

**Affiliations:** 1 Laboratory for Research in Environmental Sciences (LARES), Faculty of Agricultural Sciences, University of Chile, Santiago, Chile; 2 Research and Extension Center for Irrigation and Agroclimatology (CITRA), Faculty of Agricultural Sciences, Universidad de Talca, Campus Talca, Talca, Chile; 3 Departamento de Producción Agrícola, Facultad de Ciencias Agronómicas, Universidad de Tarapacá, Casilla 6-D, Arica, Chile; 4 Institute of Agri-Food, Animal and Environmental Sciences (ICA3), Universidad de O’Higgins, San Fernando, Chile; 5 Civil & Environmental Engineering Department, Colorado State University, Fort Collins, CO USA; 6 Center for Agricultural Water Research in China, China Agricultural University, Beijing, 100083 China; 7 Department of Agricultural Sciences, Universidad Católica del Maule, Curicó, Chile; 8 Instituto de Investigaciones Agropecuarias, INIA Carillanca, km 10 Camino Cajón-Vilcún s/n, Casilla 929, Temuco, Chile; 9 Master in Territorial Management of Natural Resources, Postgraduate School, Faculty of Agricultural Sciences, University of Chile, Santiago, Chile

**Keywords:** Climate sciences, Environmental sciences

## Abstract

Atmospheric longwave downward radiation (*L*_d_) is one of the significant components of net radiation (R_n_), and it drives several essential ecosystem processes. *L*_d_ can be estimated with simple empirical methods using atmospheric emissivity (ε_a_) submodels. In this study, eight global models for ε_a_ were evaluated, and the best-performing model was calibrated on a global scale using a parametric instability analysis approach. The climatic data were obtained from a dynamically consistent scale resolution of basic atmospheric quantities and computed parameters known as NCEP/NCAR reanalysis (NNR) data. The performance model was evaluated with monthly average values from the NNR data. The Brutsaert equation demonstrated the best performance, and then it was calibrated. The seasonal global trend of the Brutsaert equation calibrated coefficient ranged between 1.2 and 1.4, and the K-means analysis identified five homogeneous zones (clusters) with similar behavior. Finally, the calibrated Brutsaert equation improved the R_n_ estimation, with an error reduction, at the worldwide scale, of 64%. Meanwhile, the error reduction for each cluster ranged from 18 to 77%. Hence, Brutsaert’s equation coefficient should not be considered a constant value for use in ε_a_ estimation, nor in time or location.

## Introduction

Thermal atmospheric emissivity (ε_a_) is a parameter mainly used to estimate the atmospheric downward longwave radiation (*L*_d_), which is used to determine net radiation (R_n_). In this way, the R_n_ plays a significant role in modeling natural phenomena such as vegetation evapotranspiration rates, snowmelt, and frost occurrence^1^. The longwave radiation emitted by the atmosphere occurs at wavelengths between 4 and 100 μm in the electromagnetic spectrum and is influenced mainly by water vapor, carbonic anhydride, ozone, and clouds^2^.

The *L*_d_ is one of the significant components of the R_n_ model applied to forests^3^, affecting several essential ecosystem processes, such as photosynthetic rate, plant respiration, and primary productivity. However, the *L*_d_ term is not easy to measure. Although pyrgeometers are used to measure it, their high cost^4,5^ often limits their inclusion in automatic weather stations.

Then, the *L*_d_ term can be estimated using simple empirical methods adjusted based on direct measurements to solve this problem. In this regard, the literature shows that reliable estimations of *L*_d_ can be obtained using a statistical adjustment or calibration based on measured air temperature and relative humidity data measured at the surface (at screen height). However, local calibration is required^6^ for accurate results. Another approach is to use radiosonde data and atmospheric radiative transfer models, but this information limits their application to a specific date and place^7–13^.

Although an extensive database is needed, estimating ε_a_ through statistical models could be significantly improved using different parameterizations based on atmospheric conditions. This database should be able to represent a range of atmospheric conditions for every location in the world to decrease the error of statistical estimations of ε_a_.

Therefore, local calibrations of the coefficients involved in the empirical ε_a_ models are necessary. Research indicates that the majority of equations used to estimate ε_a_ are only valid for clear-sky days, reaching more accurate results when considering daily or climatological averages. Clear-sky days are defined by the absence of visible clouds in the sky. In this sense, the clear-sky conditions are defined when the clear-sky index (Global solar radiation/Extraterrestrial solar radiation) is approximately greater than 0.9^14,15^. In order to achieve this, it is possible to use existing databases such as NCEP/NCAR reanalysis to obtain more accurate estimations of the emissivity at the Earth’s surface^16,17^.

According to the literature, using semiempirical approaches to estimate ε_a_ has inherent errors linked to instrument-based measurement deviation or uncertainty. Therefore, the use of a properly calibrated ε_a_ model is a viable alternative for estimating more accurately at specific locations using meteorological variables such as air temperature and relative humidity^6,18–20^. In this regard, there is plenty of information in the literature about the estimation of R_n_ using empirical and semiempirical ε_a_ models based mainly on ground-based instruments for specific locations of different roughness surfaces worldwide^20–36^.

In this study, the performance of eight models was evaluated to determine their accuracy in the estimation of atmospheric emissivity for different locations worldwide. Climatic data from NCEP/NCAR reanalysis (NNR) were obtained from a dynamically consistent scale resolution of basic atmospheric quantities and computed parameters. Among the evaluated models, the one with the best statistical performance was calibrated on a global scale using a parametric instability analysis approach. In this way, one of the main contributions of the study at hand was to improve the R_n_ computation over homogeneous latitude areas globally, reducing the need for local calibration of atmospheric emissivity.

### Information derived from NCEP/NCAR reanalysis data (NNR)

Exploratory analysis for the NCEP/NCAR reanalysis data (1948–2020) across the world revealed that the air temperature (t_a_) varied between – 37 °C and 49 °C, with an average of 17 °C. Additionally, the actual vapor pressure (e_a_) values ranged from 0.01 to 21.9 kPa with an average value of 5.02 kPa; meanwhile, ε_a_ averaged 0.73 and varied between 0.34 and 0.97. Moreover, the variable with the most significant variation coefficient was t_a_, presenting a value of 447.7%, e_a_ with 107.6%, and ε_a_ reached an 18.5% variation.

**Figure 1:**
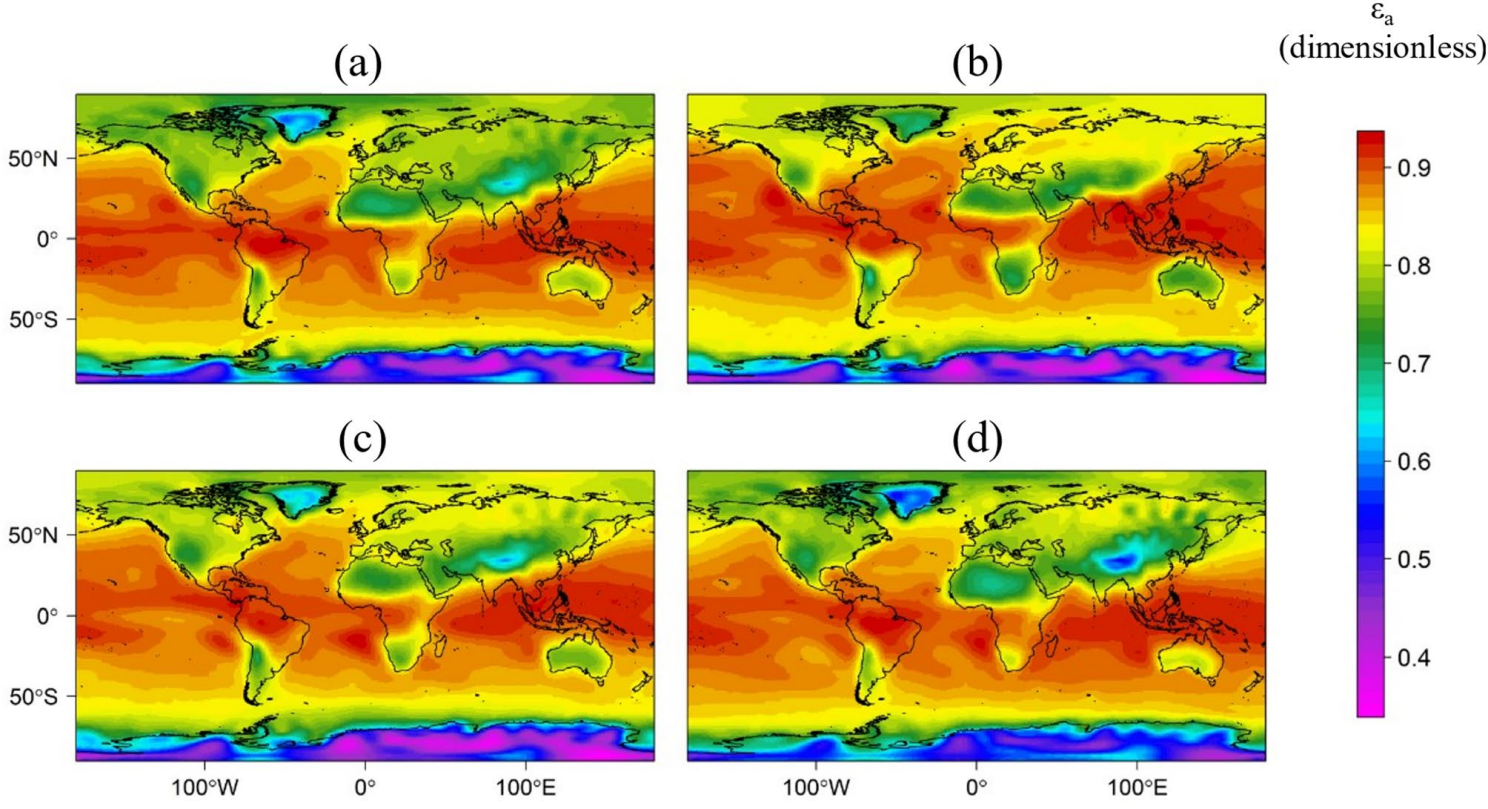
Maps of climatological world atmospheric emissivity (ε_a_) for (**a**) winter, (**b**) spring, (**c**) summer, and (**d**) autumn, calculated from NCEP/NCAR reanalysis data. This figure was obtained with R software^83^.

Figure 1 shows the observed values of ε_a_ obtained using the NNR data throughout the year’s seasons. In Fig. 1, a spatial pattern can be seen due to the formation of homogeneous groups or units (clusters) based on latitude. Moreover, this trend was also observed on a monthly scale (data not shown). These clusters have temporal variability related to atmospheric dynamics. Additionally, ε_a_ presents homogeneous values overseas and in oceans; likewise, the poles show the same trend but with different absolute values. On the continents, ε_a_ have variations related to the topography, land use, and closeness to seas and oceans. That trend was also observed in other study^37^, which indicated that uncertainties in the computation of land surface temperature, can be highly influenced by the spatial variability of the ground.

At the spatial resolution scale of the NNR, climatological variables merely correspond to a spatial trend as the NNR's spatial estimate being approximately 250 km. Comparing this data with surface weather station networks at this resolution is difficult since they correspond merely to an average in each grid element or “pixel”^38,39^. However, several studies have established the agreement between grid and ground-based observations for variables such as solar radiation, air temperature, relative humidity, precipitation, and pressure^40^. Wind speed exhibits the largest biases in space recorded, compared with other reanalysis products like ERA-40, ERA-Interim, or ERA5. Although the NNR presents significant differences, the spatial calculation resolution is different, adding additional elements, and making it challenging to make direct comparisons^38,40–44^. It is noteworthy that evapotranspiration calculated from NNR data is comparable to those calculated from observations at most weather stations^39,45^.

### Performance of atmospheric emissivity models

Descriptive analysis for the ε_a_ evaluation using the eight models is presented in Table 1. This table shows that the estimated values of ε_a_ were between 0.22 and 0.99, and the average values ranged from 0.61 to 0.83. Moreover, the Bastiaanssen model exhibited the lowest variation, with a variation coefficient of 1.1%; meanwhile, the Brutsaert model showed the highest variation coefficient, with a value of 28.1%. The other models obtained an intermediate variation with values from 6.4 to 20.1%.

**Table 1:** Descriptive statistics of estimated atmospheric emissivity (dimensionless) for each model.

Model	Minimum	Maximum	Median	Average	Standard deviation	Variation coefficient (%)
Bastiaanssen	0.71	0.76	0.76	0.75	0.0083	1.1
Prata	0.67	0.85	0.71	0.72	0.0512	7.1
Idso	0.70	0.89	0.74	0.75	0.0480	6.4
Brutsaert	0.22	0.85	0.66	0.61	0.1701	28.1
Idso and Jackson	0.74	0.99	0.82	0.83	0.0737	8.9
Swinbank	0.37	0.89	0.68	0.66	0.1330	20.1
Brunt	0.61	0.83	0.69	0.69	0.0627	9.1
Angstrom	0.65	0.82	0.72	0.72	0.0602	8.3

Table 2 presents the performance of the eight models, showing that the minimum and maximum values of the root mean square error (RMSE) were 0.097 and 0.216, respectively. Meanwhile, the coefficient of determination (r^2^) ranged from 0.45 to 0.69, while the Akaike information criterion (AIC) presented a minimum value of − 2,354,713 and a maximum of − 1,745,909. Considering all statistical parameters such as systematic error (BIAS), mean absolute error (MAE), RMSE, normalized RMSE (nRMSE), coefficient of determination (r^2^), index of agreement (d) and AIC, the Idso and Jackson model presented the poorest performance (BIAS = 0.097, MAE = 0.143, RMSE = 0.216, nRMSE = 34%, r^2^ = 0.45, d = 0.25, and AIC = − 1,745,909), while the ε_a_ Brutsaert model had the best performance (BIAS = − 0.127, MAE = 0.128, RMSE = 0.152, nRMSE = 23.9%, r^2^ = 0.76, d = 0.80, and AIC = − 2,354,713).

**Table 2:** Comparative statistics for the performance of the eight models for estimating atmosphere emissivity, using processed NCEP/NCAR reanalysis data.

Model	BIAS	MAE	RMSE	nRMSE	r^2^	d	P	AIC
Bastiaanssen	0.019	0.099	0.131	20.6	0.50	0.23	**	− 1,996,608
Prata	− 0.011	0.084	0.103	16.2	0.57	0.68	**	− 1,949,235
Idso	0.020	0.076	0.105	16.6	0.59	0.65	**	− 1,973,122
Brutsaert	0.127	0.128	0.152	23.9	0.69	0.80	**	− 2,354,713
Idso and Jackson	0.097	0.143	0.216	34.0	0.45	0.25	**	− 1,745,909
Swinbank	− 0.070	0.089	0.108	17.0	0.66	0.85	**	− 2,058,430
Brunt	− 0.041	0.088	0.101	16.0	0.64	0.75	**	− 2,058,430
Angstrom	− 0.010	0.079	0.097	15.3	0.60	0.74	**	− 2,018,959

BIAS, MAE and RMSE are the systematic error, mean absolute error, and root mean square error, respectively. The units are dimensionless. nRMSE is the normalized root mean square and corresponds to a percentage. *r*^2^ is the coefficient of determination, and *d* is the index of agreement (dimensionless). The AIC is the Akaike information criterion (dimensionless).

### Global calibration for the best model

The better performance of the Brutsaert model for estimating ε_a_ simplifies the global calibration process, considering that only one parameter remains dependent, so that the exponent’s hypothesis is invariant.

Supplementary Fig. 1 shows the seasonal behavior of ε_a_, revealing a consistent linear and positive regression with (e_a_/t_a_), independent of the season. The plot shows two separated data tendencies during the winter season with the upper right side of the graph being the most important as it concentrates more points over a linear trend.

Only a trend was evident for the spring season, with a higher concentration of points from 0.7 to 1.0. For the summer months, the graph presents the most irregular linear regression of all seasons, with the cluster of points concentrated in the top portion of Supplementary Fig. 1c, in a range of 0.8 to 1.0.

Finally, in the autumn season, two linear regressions can be identified with different slopes. However, the most important trend is located in the upper portion of the graph, where ε_a_ are concentrated in a range from 0.8 to 1.0.

The performance summary of the parametric instability analysis through the geographically weighted regression (GWR) of the spatial variation for Brutsaert equation parameters, is presented in Supplementary Table 1. The negative BIAS values show that the GWR coefficients underestimated the spatial variability of the Brutsaert equation parameters. Furthermore, the BIAS depicted a random behavior. The monthly mean RMSE was approximately 0.022 (dimensionless) with an estimation error of 1.5%. The RMSE values for autumn and winter were above the mean, while the RMSE values for spring and summer were below the mean.

As a result, the months that were closer to the average maximum temperature showed a more accurate estimation of the parameters in the Brutsaert equation compared to the colder months near the average minimum temperature. AIC values showed a similar pattern to RMSE; thus, warmer months resulted in a better AIC value than colder months. The Nash–Sutcliffe efficiency (NSE) index, d, and r^2^ indices had values near 1, indicating a good fit of the GWR coefficients for each month.

**Figure 2:**
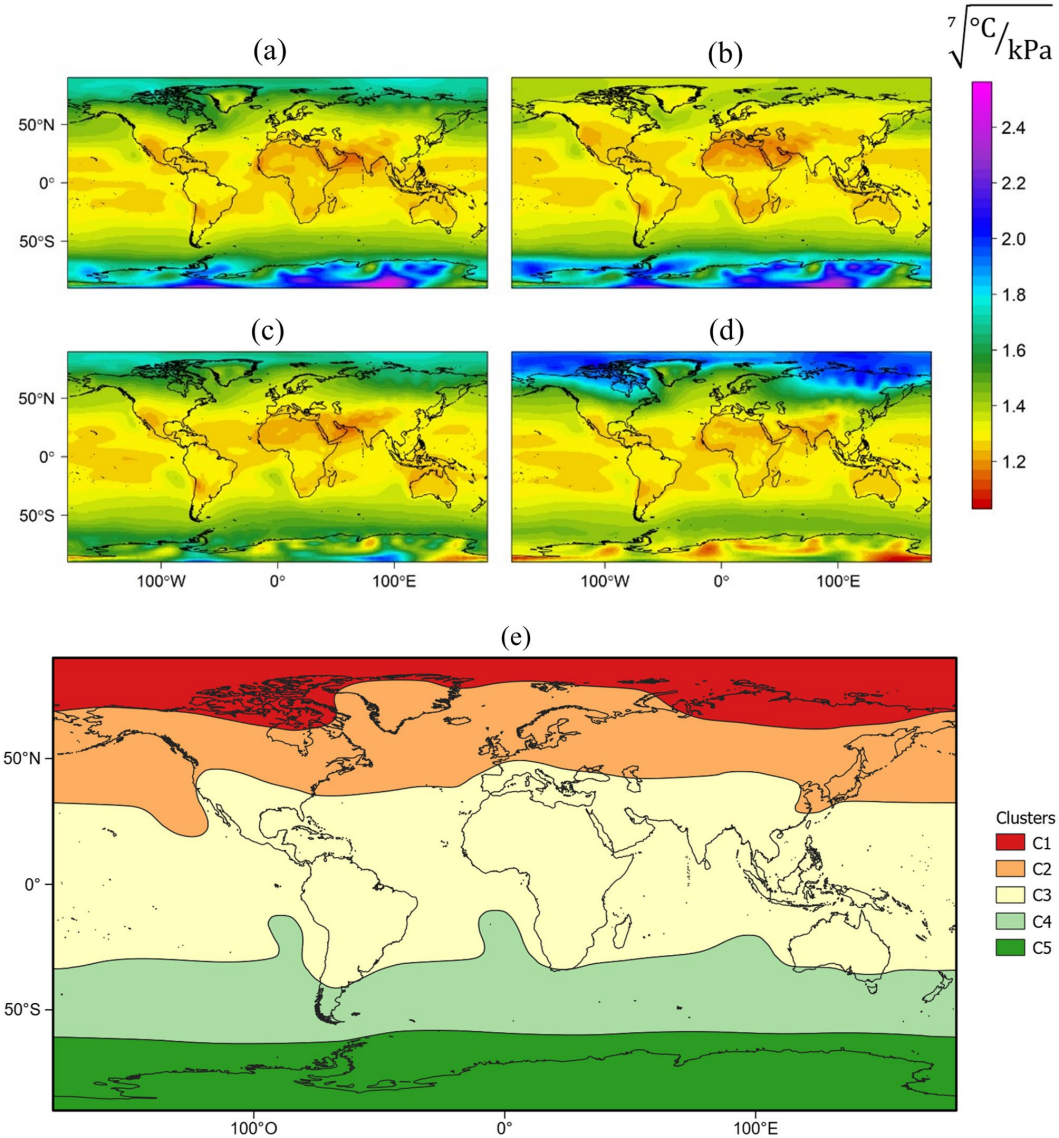
World spatial distribution of the calibrated coefficient of Brutsaert for (**a**) winter, (**b**) spring, (**c**) summer, and (**d**) autumn. Additionally, the homogeneous areas or clusters are presented (**e**), referenced for the Northern Hemisphere. This figure was obtained with R software^83^.

Figure 2 shows the global seasonal trend of calibrated Brutsaert model coefficient and the cluster resulting from the K-means analysis. The calibrated coefficient value ranged between 1.2 and 1.4, considering the four seasons and the five zones with similar behavior. In this sense, the Brutsaert model coefficient did not present a unique value for the entire world. The predominant zone was related to the Ecuador line, which covered a critical zone of the study area.

Additionally, the austral and boreal zones presented differentiation from the rest of the world. The monthly mean empirical coefficient of the Brutsaert model is shown in Supplementary Table 2. Moreover, the performance of the uncalibrated and calibrated Brutsaert equations in computing net radiation for each cluster is presented in Table 3. Here, a RMSE reduction was observed at a worldwide scale of 64%, while in Cluster 2, a RMSE decrease of approximately 77% was observed. However, Cluster 3, which mainly represents a significant portion of the land, only reached an RMSE reduction of 18%.

**Table 3:** Comparison of statistical indices for evaluating the Brutsaert equation effect on net radiation computation.

Zone	Model	BIAS (W m^–2^)	MAE (W m^–2^)	RMSE (W m^–2^)	nRMSE (%)	NSE	d	r^2^
Cluster 1	Uncalibrated Brutsaert’s	– 37.5	37.5	40.1	46.4	0.78	0.95	0.99
	Calibrated Brutsaert’s	− 4.5	6.1	7.5	8.7	0.99	1.00	1.00
Cluster 2	Uncalibrated Brutsaert’s	− 26.9	27.1	30.9	36.3	0.87	0.97	0.99
	Calibrated Brutsaert’s	− 2.3	5.5	7.0	8.2	0.99	1.00	0.99
Cluster 3	Uncalibrated Brutsaert’s	− 3.8	9.1	11.4	22.5	0.95	0.99	0.96
	Calibrated Brutsaert’s	1.7	7.2	9.3	18.2	0.97	0.99	0.97
Cluster 4	Uncalibrated Brutsaert’s	− 16.9	17.1	20.1	25.8	0.93	0.98	0.99
	Calibrated Brutsaert’s	7.6	9.0	10.6	13.6	0.98	1.00	0.99
Worldwide	Uncalibrated Brutsaert’s	− 15.2	18.1	23.6	32.0	0.90	0.98	0.97
	Calibrated Brutsaert’s	− 0.2	6.5	8.4	11.3	1.00	1.00	0.99

BIAS, MAE, and RMSE are the systematic error, mean absolute error, and root mean square error, respectively. The nRMSE is the normalized root mean square error, and its unit is %. The NSE is the Nash–Sutcliffe model efficiency index, d is the index of agreement, and r^2^ is the coefficient of determination (dimensionless). The information is only presented for four out of the five clusters.

It is important to note that the spatial resolution of the model used is approximately 250 km, which allows the calculation of meteorological variables that correspond to large climatic regions across the Earth.

The spatial configuration of the variables is influenced by factors such Earth's topography, oceans, and land surface cover, which affects variables such as albedo and surface emissivity. The Seasonal dependence is strongly associated with the Earth's trajectory in its solar orbit, affecting the incident energy of short and long waves, adjusting to the solar declination^29,46,47^.

### Improvements of estimated net radiation

**Figure 3:**
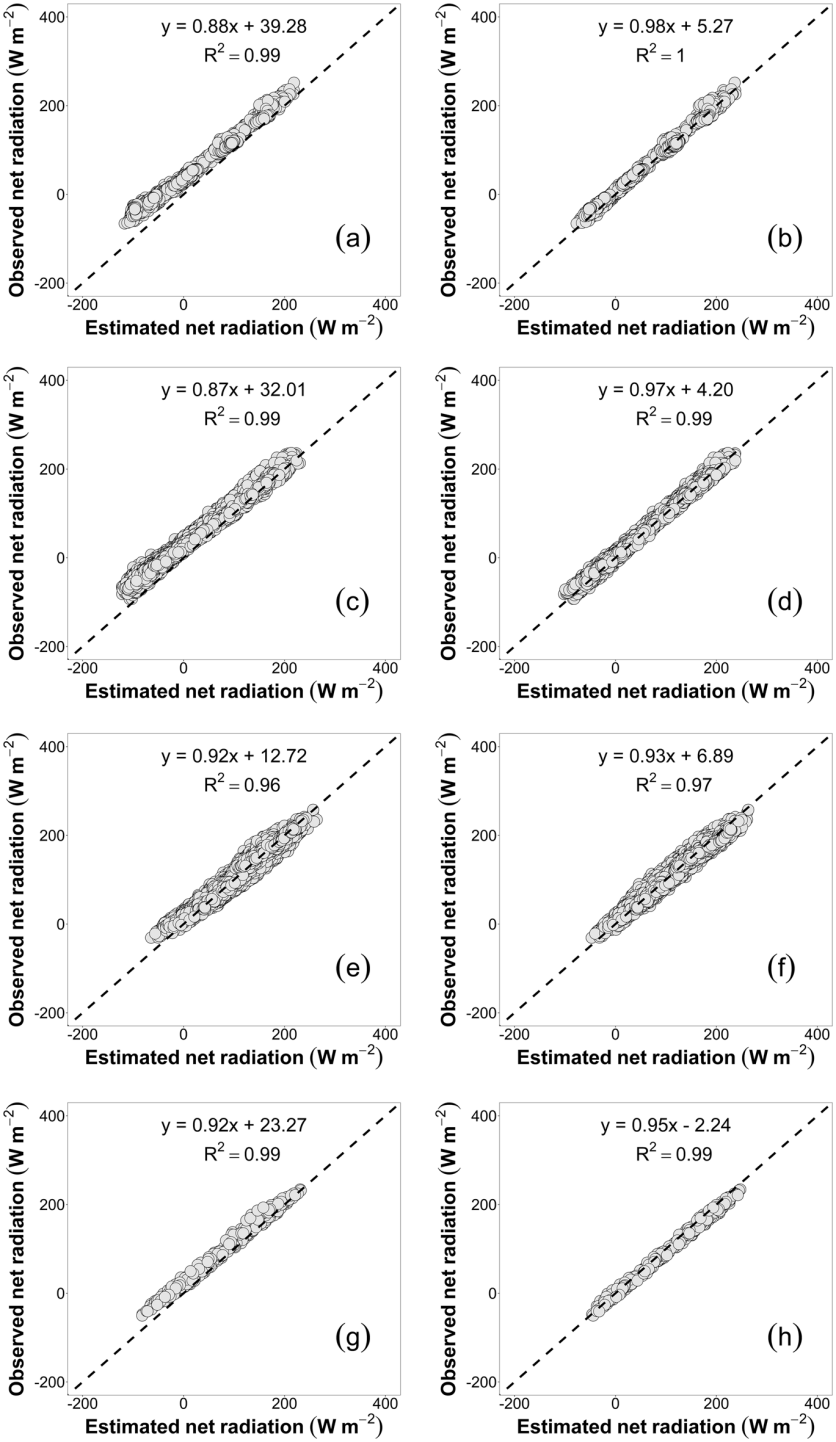
Comparison of net radiation computed using uncalibrated (**a**,**c**,**e**,**g**) and calibrated (**b**,**d**,**f**,**h**) Brutsaert’s equation in the estimated worldwide clusters: Cluster 1 (**a**,**b**); Cluster 2 (**c**,**d**); Cluster 3 (**e**,**f**); and Cluster 4 (**g**,**h**).

In high latitudes (C1 in Fig. 2), a calibrated Brutsaert model coefficient demonstrated good agreement between the observed and estimated values of R_n_ (Fig. 3b) values using a calibrated Brutsaert model coefficient (Cluster 1 in Table 3). Low error values were observed for BIAS, MAE, and RMSE, − 4.5, 6.1, and 7.5 W m^–2^, respectively.

At the same time, the NSE, d, and the r^2^ had values close to 1.0 and a value of nRMSE equal to 8.7%. In this context, estimated R_n_ for latitudes greater than 60° N under all-sky conditions from MODIS imagery, was validated using data from 82 sites and eight different observation networks^27^.

These authors found acceptable accuracy values of RMSE ranging between 15.04 and 23.66 W m^–2^, whereas the values of r^2^ and BIAS were between 0.51 and 0.85 and between − 0.08 and 0.27 W m^2^, respectively. However, for Alaska’s Arctic tundra summer conditions (at USA sites Fen, Tussock, and Heath, the latitude of 68° N), the estimated R_n_ for all-sky conditions aligned well with the observed values, presenting an average RMSE of 23 W m^–2^ and r^2^ value equal to 0.99 using the remote sensing thermal-based two-source energy balance model^48^.

Errors were found using a similar value for the original Brutsaert coefficient (1.25 ± 0.009), which was consistent with our study for summer condition (S Table 1). When estimating the R_n_ in the geographic areas corresponding to Cluster 2 (Table 3), latitudes are between 40 and 60°N (C2 in Fig. 3), and lower errors were observed, with values of 49, 10, and 7% for BIAS, MAE and RMSE, respectively. Simulations of R_n_ in different zones, locations, and vegetation surfaces have been performed within a range of latitudes^34^, 41–60°N in Canada (Eloria, Ontario, with a latitude of 43°N) using an empirical R_n_-Model observed an average MAE, r^2^ and d of approximately 28 W m^–2^, 0.98 and 0.99, respectively. At the same latitude but different locations in Avignon, France^30^ (latitude 43°N), the R_n_ was evaluated using a semiempirical model based on Stefan–Boltzmann under grass cover, observing a RMSE of 34 W m^-2^ with a calibrated Brutsaert’s coefficient of 1.31.

Also, the R_n_ has been estimated for clear-sky and all-wave net radiation combined visible and shortwave infrared (VSWIR) and thermal infrared (TIR) remote sensing data at a location in Montana, USA (Fort Peck, latitude 48° N)^49^, observing that the component-based approach presented a BIAS, RMSE and r^2^ of 76.7, 2.0 and 0.87, respectively. Using a direct estimation approach, the BIAS, RMSE, and r^2^ values were 52.3, − 1.5, and 0.94, respectively. Furthermore, R_n_ estimations from solar shortwave radiation measurements and conventional meteorological observations (or satellite retrievals) were conducted at 24 different sites^50^. Three of these sites were located at latitudes over 42° N (Fort Peck, MT in grass cover; Sioux Falls, SD in grass cover; and Wind River, WA in temperate evergreen forest cover). Thus, the errors obtained at those sites were 17.2, 20.8, and 16.0 W m^–2^ for RMSE and 3.5, 2.6, and 4.6 W m^–2^ for BIAS, respectively. For mid-latitudes (C3 in Fig. 3), the estimation of R_n_ (Fig. 3f), using a calibrated Brutsaert’s coefficient (Cluster 3 in S Table 2), presented a statistical mean deviation lower than 9.3 W m^–2^. The evaluation of the different R_n_ models presented in the literature worldwide are mainly inserted between latitudes 42°N and 40°S. In this context, R_n_ was estimated using MODIS data for clear sky days with an empirical ε_a_ equation^51,52^, and this study covered most of Oklahoma and the southern part of Kansas, USA (latitude from approximately 34.5°to 38.5°N and longitude from 95.3° to 99.5°W).

Thus, the comparison between observed and simulated R_n_ presented 59 W m^–2^, 74 W m^–2^, and 0.89 for BIAS, RMSE, and r^2^, respectively. On the other hand, for the climate of Baghdad, Iraq (latitude 33°N) in natural prairie grass, there was a good agreement between observed and estimated R_n_ with a simple empirical approach for all clear skies^53^, with an average RMSE value equal to 28 W m^–2^ and r^2^ value of 0.984.

However, in a semiarid climate covered by sparse vegetation near Tabernas^54^, Almería, Spain (37°N) good agreement was obtained between the observed and simulated R_n_, with a mean RMSE value of 47 W m^–2^ and r^2^ value of 0.96. In another study conducted on an olive vegetation surface in southwestern Sicily, Italy (37°N)^55^, an RMSE value of 35.4 W m^–2^ was obtained in the R_n_. They used a semiempirical model based on the Stefan–Boltzmann law that included estimating longwave radiation incorporating the original Brutsaert’s coefficient value. Furthermore, in a wetland in the Paynes Prairie Preserve (29° N) in north-central Florida, USA^56^, the R_n_ was estimated using GOES satellite data. Thus, the R_n_ was best characterized when the GOES solar and GOES longwave radiation products were combined, reaching average RMSE, NSE, and r^2^ values equal to 14.1 W m^–2^, 0.92, and 0.95, respectively.

In another experience, in the Upper Blue Nile Basin, Ethiopia (7.5°–12.5° N)^57^ the R_n_ distribution was estimated from satellite MODIS and automatic weather station, obtaining a reduction in the mean bias (MB) and RMSE values of 76.43 and 17.71%, respectively, by implementing the new recalibrated Brutsaert equation.

Furthermore, the solar shortwave radiation data and meteorological observations (or satellite retrievals) from 24 different sites (USA and China) were used to estimate R_n_^50^, where 21 of them were in latitudes between 32° and 41°N under various land covers (grass, pastures, wheat, rangeland, crop, forest, native prairie, and desert). Across all sites and land covers, the BIAS varied between 19.7 and 27.8 W m^–2^, while the RMSE ranged from 12.8 to 21.0 W m^–2^.

For grass cover, a semiempirical model based on the Stefan–Boltzmann law was evaluated to estimate R_n_ in Talca, Chile (35°S)^30^, obtaining an RMSE of 42 W m^–2^ with a calibrated Brutsaert’s ε_a_ coefficient equal to 1.31. Furthermore, for olive tree cover (Pencahue Valley site, Pencahue, Chile, latitude 35°S and CIFA Experimental Station site, Córdoba, Spain, latitude 37.8°N) and vineyard cover (Pencahue Valley site, Pencahue, Chile, latitude 35°S), the estimation of R_n_ was observed with RMSE and MAE values below 45.0 and 31.0 W m^–2^, respectively^25,35,36^. For latitudes from 41°S to 60°S (C4 in Fig. 3), the estimation of R_n_ (Fig. 3h), using a calibrated Brutsaert’s coefficient (Cluster 4 in Table 3), presented a statistical mean deviation lower than 10.6 W m^–2^.

In this case, there is limited literature about the estimation of R_n_ in southern latitudes. Thus, the approaches presented in this study are promising for improving the estimation of R_n_ by incorporating a calibrated Brutsaert’s ε_a_ equation coefficient, broken down by homogeneous latitudes separated into five zones around the world (Figs. 2e and 3). Additionally, the errors found in this study are lower and similar in the same case compared to those found in the existing literature. In this sense, it is necessary to further evaluate ε_a_ estimates under a larger number of land cover types, different vegetation architectures of surface roughness lengths, and other characteristics than the Brutsaert ε_a_ equation coefficient values obtained in this research.

## Conclusions

A spatially explicit approximation method for calculating atmospheric emissivity (ε_a_) has been investigated to improve the estimation of downward longwave radiation during the day and further enhance radiation calculations.

The study evaluated eight models globally to estimate air emissivity using the NCEP/NCAR Reanalysis database, which corresponds to spatial trends in each variable used, mainly due to the calculation resolution, which is 2.5° in latitude and longitude. The results showed that the Brutsaert ε_a_ model had the best performance (BIAS = − 0.127, MAE = 0.128, RMSE = 0.152, nRMSE = 23.9%, r^2^ = 0.76, d = 0.80, AIC = – 2,354,713), and it was calibrated using geographically weighted regression (GWR) for global use.

The calibrated values considerably improved the calculation of the components of the surface energy balance, reducing calculation errors in net radiation from 25.2 W m^–2^ (nRMSE = 32.6%) to 8.6 W m^–2^ (nRMSE = 12.0%). The study indicates that the Brutsaert ε_a_ model should not be considered to have a constant coefficient value in time or space. It is advisable to use the coefficients found in this work to minimize errors when calculating net radiation. Using a sinusoidal equation or spline-type interpolation to reproduce the temporal variability of the coefficients for each day of the year is recommended when using the average monthly coefficients of the Brutsaert equation to estimate the emissivity of the atmosphere at a daily level.

## Methods

Due to the different climatic conditions, the entire world was used as the study area to achieve an adequate model evaluation and calibration. Observed climatic data were obtained from a dynamically consistent scale resolution of basic atmospheric quantities and computed parameters known as NCEP/NCAR reanalysis data (NNR). These data were produced by the US National Centers for Environmental Predictions (NCEP) and the National Center for Atmospheric Research (NCAR) based in Boulder, CO, USA^16^.

### NCEP/NCAR reanalysis data

The NNR data of global climatic information cover the period from 1948 to the present. Its spatial resolution is 2.5° longitude and 2.5° latitude with a temporal resolution of one month, one day, or six hours, and diagnosed diabatic heating of 17 vertical isobaric levels from 1000 to 10 hPa^58–60^. The NNR data were developed by the synergy of processes such as quality control, assimilation, interpolation, observed data acquired by ground and sea stations, planes, satellites, and atmospheric soundings, together with simulations of atmospheric general circulation models using the Climate Data Assimilation System (CDAS)^58^.

The data used in this research were based on the “Surface” and “Surface flux” sections and their upward solar radiation flux.

### Atmospheric emissivity parameterizations

Below are the equations used to estimate ε_a_ with meteorological variables such as t_a_ and actual vapor pressure (e_a_). The exception is the Bastiaanssen model^21^ because it estimates ε_a_ at a daily scale for any condition of cloudiness, only depending on atmospheric transmissivity (τ_sw_). The Bastiaanssen model was calibrated^61^ and used in the satellite-based energy balance for mapping evapotranspiration with an internalized calibration (METRIC) model^62^. The eight evaluated models are the following^2,12,13,21,52,63–65^:

$${\varepsilon }_{a}=\text{0.85}{\left(-{\text{Ln}}\left({\tau }_{sw}\right)\right)}^{0.09},$$

$${\varepsilon }_{a}=1-\left(1+\xi \right)\cdot {e}^{-\sqrt{\text{1.2}+\text{3.0}\cdot \xi }},$$

$${\varepsilon }_{a}=\text{0.70}+\text{0.0000595}\cdot {e}_{a}\cdot {e}^{\left({1500}/{T}_{a}\right)},$$

$${\varepsilon }_{a}=1.24\cdot {\left({e}_{a}/{T}_{a}\right)}^\frac{1}{7},$$

$${\varepsilon }_{a}=1-\text{0.261}\cdot {e}^{-\text{0.000777}\cdot ({273}-{T}_{a}{)}^{2}},$$

$${\varepsilon }_{a}=\text{0.0000092}\cdot {\left({T}_{a}\right)}^{2},$$

$${\varepsilon }_{a}=\text{0.605}+\text{0.048}\cdot \sqrt{{e}_{a}},$$

$${\varepsilon }_{a}=\text{0.83}-\text{0.18}\cdot {1}{\text{0}}^{-\text{0.067}\cdot {e}_{a}},$$

where ε_a_ is the atmospheric emissivity (dimensionless) for clear-sky conditions based on air temperature (T_a_, K), water vapor pressure (e_a_, hPa), atmospheric transmissivity (τ_SW_, dimensionless), and altitude (z, meters above sea level). The z was obtained from the WorldClim data^66^ with a spatial resolution of 1 km. Moreover, *e*_a_ was estimated as follows^29,67^:

$${e}_{a}=6.108\cdot \left(\frac{RH}{100}\right)\cdot {e}^{\left(\frac{17.27\cdot {t}_{a}}{{t}_{a}+237.3}\right)},$$

where e_a_ is the actual water vapor pressure (hPa), t_a_ is the air temperature (°C), and RH is the relative humidity (%). Also, the τ_SW_ and ξ were calculated according to:

$${\tau }_{\text{sw}}=\text{0.75}+\text{0.00002}\cdot z,$$

$$\xi =\text{46.5}\cdot \left({e}_{a}/{t}_{a}\right),$$

The observed values of ε_a_ were calculated as follows:

$${\upvarepsilon }_{\mathrm{a}}=\frac{{L}_{d}}{\upsigma \cdot {\mathrm{T}}_{\mathrm{o}}^{4}},$$

where T_o_ corresponds to the average temperature of the whole air profile (K) measured by a meteorological station, and *L*_d_ is the atmospheric longwave downward radiation (W m^–2^).

For this study, the T_o_ and *L*_d_ data was obtained from the reanalysis databases. On the other hand, the estimated values of ε_a_ from the eight models were calculated using t_a_ and RH obtained from the same reanalysis databases.

The evaluation of the goodness of fit for each model was conducted using the monthly average values of the NCEP/NCAR reanalysis (NNR) data.

### Statistical analysis

The evaluation of the goodness of fit for each model was conducted using the monthly average values of the NNR data through the determination of the systematic error^68^ (BIAS), mean absolute error^68^ (MAE), root mean square error^68^ (RMSE), normalized root mean square error^69^ (nRMSE), and coefficient of determination^70^ (r^2^) (Table 3). Additionally, the index of agreement (d) was used^70–75^, as well as the Akaike information criterion (AIC)^76–78^. Also, the Nash–Sutcliffe efficiency (NSE) index^79^ was used, and it can range from − ∞ to 1. An efficiency of 1 (NSE = 1) corresponds to a perfect match of modeled data to the observed data. An efficiency of 0 (NSE = 0) indicates that the model predictions are as accurate as the mean of the observed data, whereas an efficiency less than zero (NSE < 0) occurs when the observed mean is a better predictor than the model. Essentially, if the model efficiency is closer to 1, the model is more accurate. NSE is equivalent to the coefficient of determination (r^2^), thus ranging between 0 and 1.

### Global calibration

The ε_a_ model with the best performance was adjusted globally using geographically weighted regression (GWR). For this analysis, the dependent variable was ε_a_, and the independent variables were e_a_ and T_a_. The GWR was carried out using NNR data, where information on each pixel was extracted from the grids, generating a vector type point layer for every month.

The GWR is based on weighted least squares^80^, considering the distance between each point, and it is described with the following equation^80–82^:

$${y}_{i}={a}_{0}\left({u}_{i},{v}_{i}\right)+{\sum }_{k}{a}_{k}\left({u}_{i},{v}_{i}\right){x}_{ik}+{\delta }_{i},$$

where (u_i_, v_i_) corresponds to the coordinates of the ith point in the space, y_i_ is the dependent variable value, x is the kth independent variable in the ith point, a_0_ and a_k_ are the regression parameters in the ith point, and δ_i_ is the error in the ith point. The a_k_(u_i_, v_i_) coefficients were estimated as follows:

$${a}_{k}\left({u}_{i},{v}_{i}\right)={\left[{X}^{T}\cdot W\left({u}_{i},{v}_{i}\right)\cdot X\right]}^{-1}\cdot {X}^{T}\cdot W\left({u}_{i},{v}_{i}\right)\cdot Y,$$

where the dependent and independent variables are in the Y and X matrices, respectively.

All calculations, statistical analyses, and figures were processed using R software^83^ and the libraries “raster”^84^, “rgdal”^85^, “hexbin”^86^, “hydroGOF”^87^, “topmodel”^88^, and “GWmodel^89,90^.

### Net radiation improvements

R_n_ was computed at a global scale to evaluate the impact of the ε_a_ calibrated model on the traditional method of calculating net radiation. The R_n_ is the sum of downward (incoming) and upward (outgoing) shortwave and longwave radiation, which is also a measure of the available energy at an underlying surface. It is also the fundamental parameter that governs the climate of the planetary boundary layer and is the driving force for processes such as evapotranspiration, air and soil heating, and photosynthesis. The net radiation over the terrestrial surface can be calculated as follows^21^:

$${R}_{n}=\left(1-\alpha \right){R}^{\downarrow }+{L}^{\downarrow }-{L}^{\uparrow }-\left(1-{\varepsilon }_{s}\right){L}^{\downarrow },$$

where R_n_ is the estimated net radiation (W m^-2^); R^↓^ is the downward shortwave solar radiation (W m^–2^); L^↓^ and L^↑^ are the downward and upward longwave radiation, respectively (W m^–2^); α is the surface albedo (dimensionless); and ε_s_ is the surface emissivity (dimensionless). The components of the incoming and outgoing longwave radiation, respectively, are given by:

$${L}^{\downarrow }={\varepsilon }_{a}\sigma {T}_{a}^{4},$$

$${L}^{\uparrow }={\varepsilon }_{s}\sigma {T}_{s}^{4},$$

where ε_a_ is atmospheric emissivity (dimensionless); T_a_ is air temperature (K); T_s_ is the land surface temperature (K), which was obtained from the monthly mean MOD11C3 product^91^; and σ is the Stefan–Boltzmann constant (5.67 × 10^–8^ W m^–2^ K^–4^).

ε_s_ can be calculated from a simple linear regression using the normalized difference vegetation index or NDVI^92^, which is necessary to estimate the land surface temperature (LST). The values of ${\upvarepsilon }_{\mathrm{s}}$ were calculated as follows^25^:

$${\varepsilon }_{s}=0.9585+0.0357\cdot NDVI,$$

where the NDVI is obtained from the monthly mean MOD13A3 product^93^.

ε_a_ is determined according to Brutsaert’s^64^ method, where the observed R_n_ was computed with the shortwave and longwave radiation from the NCEP/NCAR reanalysis. Meanwhile, the estimated R_n_ was obtained using the uncalibrated and calibrated parameters of Brutsaert’s equation and calculating the longwave radiation using ${L}^{\downarrow }$ and ${L}^{\uparrow }$ equations.

### Supplementary Information

Supplementary Information.

## Data Availability

The datasets used and/or analysed during the current study available from the corresponding author on reasonable request.
