# Supplementary material for: Assessment of atmospheric emissivity models for clear-sky conditions with reanalysis data
Source: Sci Rep. 2023 Sep 2;13:14465. doi: 10.1038/s41598-023-40499-6 (PMC10475081; doi:10.1038/s41598-023-40499-6)
Supplement: Supplementary file 1 — Supplementary Information. [file 41598_2023_40499_MOESM1_ESM.docx]

**SUPPLEMENTARY INFORMATION**


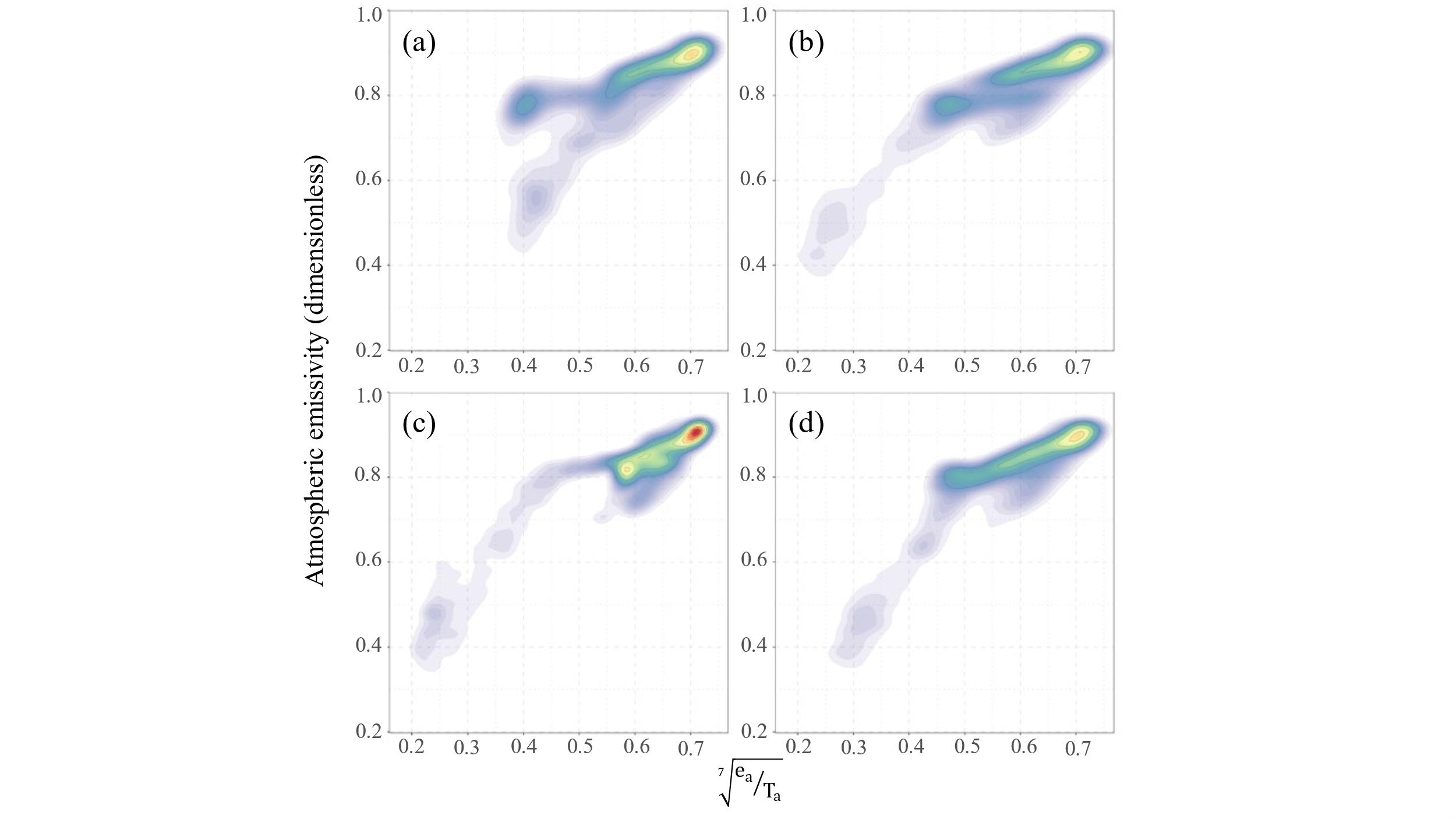


**Supplementary Fig. 1**. Scatter plot for atmospheric emissivity vs. (e_a_/T_a_)^1/7^ for (a) winter, (b) spring, (c) summer, and (d) autumn referenced for the Northern Hemisphere, calculated from NCEP/NCAR reanalysis data.

**Supplementary Table 1.** Summary statistics of geographically weighted regression coefficients for the estimation of the spatial variation of Brutsaert equation coefficients.

| Month | m(x,y) | BIAS | RMSE | NSE | d | AIC | r^2^ | p^1^ |
| --- | --- | --- | --- | --- | --- | --- | --- | --- |
| January | 1.428 ± 0.219 | -2.1x10^-5^ | 0.0171 | 0.9939 | 0.97 | -56,739 | 0.99 | ** |
| February | 1.438 ± 0.212 | -1.5x10^-4^ | 0.0195 | 0.9915 | 0.97 | -53,935 | 0.99 | ** |
| March | 1.459 ± 0.221 | -1.7x10^-4^ | 0.0227 | 0.9894 | 0.97 | -50,689 | 0.99 | ** |
| April | 1.444 ± 0.217 | -5.3x10^-5^ | 0.0248 | 0.9869 | 0.97 | -49,065 | 0.99 | ** |
| May | 1.419 ± 0.209 | -1.2x10^-4^ | 0.0256 | 0.9849 | 0.96 | -48,337 | 0.99 | ** |
| June | 1.406 ± 0.207 | -1.9x10^-4^ | 0.0262 | 0.9840 | 0.96 | -47,783 | 0.99 | ** |
| July | 1.406 ± 0.202 | -2.2x10^-4^ | 0.0269 | 0.9823 | 0.96 | -47,315 | 0.98 | ** |
| August | 1.404 ± 0.193 | -2.5x10^-4^ | 0.0265 | 0.9811 | 0.96 | -47,707 | 0.98 | ** |
| September | 1.410 ± 0.177 | -3.3x10^-4^ | 0.0245 | 0.9808 | 0.96 | -49,275 | 0.98 | ** |
| October | 1.398 ± 0.139 | -4.7x10^-4^ | 0.0208 | 0.9775 | 0.96 | -52,899 | 0.98 | ** |
| November | 1.399 ± 0.175 | -2.5x10^-4^ | 0.0179 | 0.9895 | 0.96 | -55,930 | 0.99 | ** |
| December | 1.418 ± 0.203 | -4.4x10^-5^ | 0.0164 | 0.9934 | 0.97 | -57,605 | 0.99 | ** |

^1.^ ** p < 0,0001; BIAS, MAE, and RMSE are the systematic error, mean absolute error, and root mean square error, respectively. The units are dimensionless. The nRMSE is the normalized root mean square error, and its unit is %. The NSE is the Nash–Sutcliffe model efficiency coefficient, d is the index of agreement, and r^2^ is the coefficient of determination (dimensionless).

**Supplementary Table 2.** Monthly mean values for the empirical coefficient of the Brutsaert model for each cluster.

| Cluster | Jan | Feb | Mar | Apr | May | Jun | Jul | Aug | Sep | Oct | Nov | Dec | Avg |
| --- | --- | --- | --- | --- | --- | --- | --- | --- | --- | --- | --- | --- | --- |
| C1 | 1.941 | 1.942 | 1.882 | 1.645 | 1.444 | 1.361 | 1.376 | 1.398 | 1.506 | 1.665 | 1.809 | 1.891 | 1.655 |
| C2 | 1.584 | 1.559 | 1.497 | 1.426 | 1.369 | 1.338 | 1.329 | 1.332 | 1.361 | 1.419 | 1.498 | 1.558 | 1.439 |
| C3 | 1.283 | 1.279 | 1.274 | 1.270 | 1.270 | 1.270 | 1.271 | 1.271 | 1.270 | 1.271 | 1.276 | 1.282 | 1.274 |
| C4 | 1.382 | 1.383 | 1.386 | 1.392 | 1.400 | 1.409 | 1.418 | 1.419 | 1.413 | 1.404 | 1.394 | 1.388 | 1.399 |
| C5 | 1.335 | 1.426 | 1.661 | 1.801 | 1.827 | 1.825 | 1.813 | 1.785 | 1.729 | 1.500 | 1.333 | 1.323 | 1.613 |
